# Supplementary material for: The effects of Alcohol Dependence on the CSF Proteome in Mice: Evidence for Blood-Brain Barrier Dysfunction and Neuroinflammation
Source: bioRxiv. 2025 Nov 12:2025.11.10.687295. Preprint. [Version 1] doi: 10.1101/2025.11.10.687295 (PMC12642327; doi:10.1101/2025.11.10.687295)
Supplement: Supplement 4 [file media-4.docx]

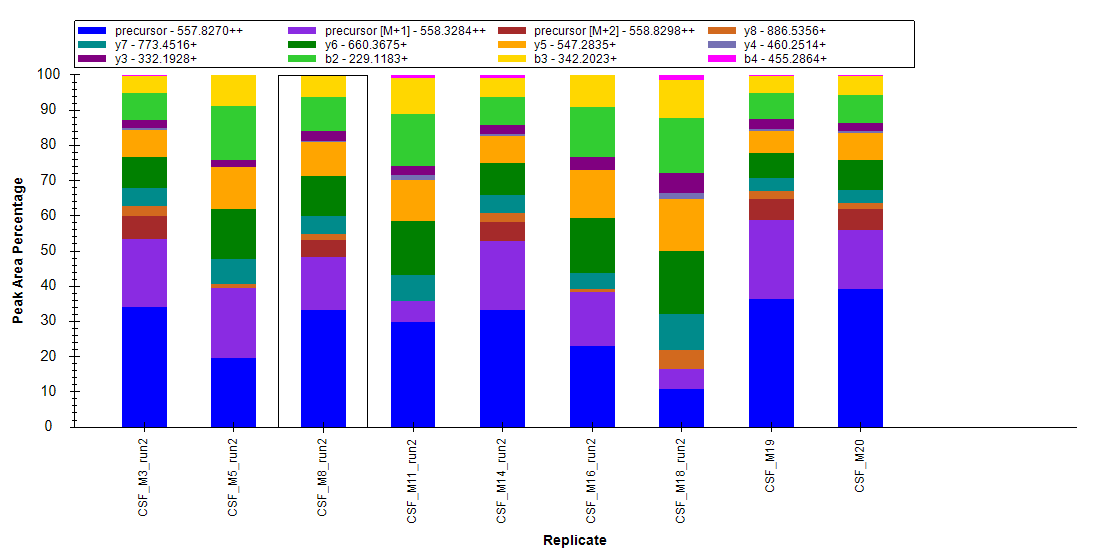


**Figure S1:** Peak area percentages of the IGG2B_RAT peptide DILLISQNAK (normalized to total peak area). Legend indicates ion types.
